# Socio-demographic, personal, environmental and behavioral correlates of different modes of transportation to work among Norwegian parents

**DOI:** 10.1186/s13690-016-0155-7

**Published:** 2016-10-10

**Authors:** Oline Anita Bjørkelund, Hanna Degerud, Elling Bere

**Affiliations:** 1 Department of Public Health, Sport and Nutrition, Faculty of Health and Sport, University of Agder, Service Box 422, 4604 Kristiansand, Norway; 2 Present address: Department of Health Science and Technology, Physical Activity and Human Performance group - SMI, Faculty of Medicine, Aalborg University, Fredrik Bajers Vej 5 Postbox 159, 9100 Aalborg, Denmark

## Abstract

**Background:**

Cycling and brisk-walking to work represents an opportunity to incorporate sustainable transport related moderate- to- vigorous physical activity (MVPA) into daily routine among adults, and thus, may make an important contributing to health. Despite the fact that walking and cycling is an option for many commuters and also brings a number of benefits, a considerable proportion of commuters choose to use other means of transport when cycling and walking would be a highly appropriate transport mode. The object of this study was to assess the associations between modes of commuting to the workplace among parental adults; taking socio-demographic, personal, environmental and behavioral factors into account.

**Methods:**

Data from a cross- sectional questionnaire were collected from a sample of 709 parents (23 % men and 77 % women) of children aged 10–12 years-old in two Norwegian counties, Hedmark and Telemark. Commuting behavior, socio- demographic determinants, personal and environmental factors were ascertained using questionnaire data from the Fruit and Vegetables Makes the Marks project (FVMM). Multivariate logistic regressions were applied.

**Results:**

In total, 70 % of adults were categorized as car commuters to and from work, 12 % was categorized as a cyclist and 7 % as a walker. The multivariate analyses showed that active commuters were more likely to have a shorter distance to work and perceived the traffic as more safe. Moreover, those who actively commute to the workplace considered commuting as a way to obtain health benefits and a way to reduce CO_2_ emissions. Active commuters also considered weather to be an obstacle to active commuting.

**Conclusion:**

In this cross-sectional study of parents living in sub-urban Norway, we found that active commuting to and from the workplace were associated with a shorter distance to work, traffic safety, environmental concern, health benefits and weather condition. In light of these findings, cycling to work seems to be the most appropriate target for interventions and public health campaigns within this population.

## Background

An active lifestyle with regular physical activity is associated with beneficial effects on a range of health outcomes [1, 2], reduced risk of chronic diseases [3, 4] and enhancement of self- reported well-being [5–7]. Cycling and brisk-walking to work represents an opportunity to incorporate sustainable transport related moderate- to- vigorous physical activity (MVPA) into daily routine among adults, and thus, may make an important contributing to health [8–10]. Accordingly, daily repetitive active transport has been reported to relate inverse with metabolic risk factors for cardiovascular disease [4, 11], prevalence of diabetes 2 [12], obesity [13–15], and positively with physical fitness [16–18].

Despite the fact that walking and cycling is an option for many commuters and also brings a number of benefits, a considerable proportion of commuters choose to use other means of transport when cycling and walking would be a highly appropriate transport mode [13, 19, 20]. Hence, trend data for high-income countries indicate that transport related physical activity has decreased in the past 20–30 years [20–22]. Clearly, active commuting has some potential disadvantages and different reasons have been suggested, such as the difficulties of carrying heavy loads, being at the mercy of the weather, traffic safety and distance [13, 19, 20]. Correspondingly, a large number of studies across different countries, for instance The Netherlands, Denmark, Germany, Belgium, UK and US, have examined the relationship between determinants and active commuting among students and adult population [13, 19, 23–26]. Some studies have found psychological factors important, such as strong habits [27–29], high self-efficacy [25], positive intensions [29] and attitudes towards active transportation [30]. Others have found influential factors in the environment, such as traffic safety [31–33], residential density, land use mix use [34, 35] and short distance between home and work [36]. However, the majority of these studies assessed either walking alone or as a pool of active commuters that include both cyclist and walkers, and thus potentially neglected which specific determinants characteristics are most important for commuters’ mode of travel. Moreover, there has been little agreement on how commuting should be measured and inconsistent measures of travel habits have been reported in previous studies with little or no information on their validity and reliability. Clearly, there is a need of studies using specific and precise measurements of active commuting.

Among developing countries, the prevalence of active travel for any purpose is highest in northern European countries where walking and cycling are far more common, than in Mediterranean cities and the United States of America (US) [12, 19, 24, 37, 38]. In general, it is also reported that the use of public transport, which normally requires walking or cycling to a station, is also more common in Europe than in US and Mediterranean countries [13, 31]. In example, the prevalence of commuter walking in the US is reported approximately 2.5–3 %, while cycling consist of 0.5–1 % of total commuter trips [12, 37]. On the other hand, countries in northern Europe, eg Denmark, Belgium and the Netherlands have much higher prevalence of active commuters, in general approximately 40–50 % of total commuter trips to work are made by either walking (20–25 % of total commuting trips) or cycling (20–25 % of total commuting trips) in these countries [21, 39, 40]. In Norway, Vågane and collegues (2012) has presented data from a study measuring usual mode of travel transportation in a national Norwegian sample and found that among 11 % of commuter trips was made by either walking or bicycling [41]. However, it is important to be aware of that comparisons of data from different countries are difficult, because no standardized method has been used in commuting and transport research [23]. Moreover, there is also major differences in active transportation habits across countries, even when geography, population density and, climate are apparently similar [20]. On the other side, there is consistent evidence across different countries that the benefits of active transport are multifactorial, and include in addition to opportunities for habitual physical activity and beneficial health effects, reduced pollution emission, less traffic, and greater social interactions [13, 20, 42]. It is also likely that active transport could represent a time- efficient, cheap and thus feasible approach for increasing levels of physical activity, [19, 30, 43] which is important, especially among working parents.

Therefore, better insight in factors associated with active commuting can provide an empirical basis for effective intervention among parents. Accordingly, the aim of this study was to assess the associations between modes of commuting to the workplace and socio-demographic, personal, environmental and behavioral factors into account among parents.

## Methods

### Research design and setting of the study

The present study is part of the project “cohort II” survey within the Fruit and Vegetables Makes the Marks project (FVMM) [44] and the Active Transportation to school and work in Norway project [26, 45]. Research clearance was obtained from the Norwegian Social Science Data Services (NSD; ID = 22405). Informed written consent was sought from all the participants.

### Characteristics of participants

The sample includes 709 parents of children in 6^th^ and 7^th^ graders (10–12-years of age) at 27 randomly selected schools in two Norwegian counties, Hedmark and Telemark. The data collection took place in September 2008 were a total of 1339 schoolchildren (out of 1912 eligible) brought home a parent questionnaire to be completed independently by one of their parents. A total of 1012 parents completed the questionnaire. Based on the answers from the 1012 questionnaires, we excluded parents not working away from home (*n* = 128) and those working less than 1 day a week away from home (*n* = 38). We also excluded parents with inconsistent or erroneous answers (*n* = 137). This included foremost a large group of participants where the number of days that they reported working away from home did not correspond to how many days a week they reported using different modes of commuting. For instance, some reported working 5 days a week away from home, but only reported commuting by any given mode of transportation for three of these days. We only included participants where this reporting was completely consistent. The final selection that was included in statistical analyses consisted consequently of 709 parents, whereas 690 of them had reported gender.

### Measures

#### Mode of commuting

Commuting to work was obtained using a questionnaire to record participant’s self-reported travel to and from work based on a questionnaire matrix shown to have acceptable test-retest reliability [45] and a significant rank order agreement [46].

Hence, the outcome was assessed with separate items to and from work for different seasons; fall, winter, spring and summer by asking; “How many days a week do you travel to/from work?: (1) walking (2) cycling (3) by car (4) by public transport, giving a total of eight responses (to and from work) per mode of commuting (ie to and from work for fall, winter, spring and summer). The number of trips for all seasons was grouped and the mean number of trips per week for walking, cycling, car commuting and public transport was calculated. Based on the average number of trips/week the parents were categorized into one specific mode of commuting if more than 50 % of the trips were conducted by that specific mode. If the mean number of trips did not count to a specific main mode of commuting (>50 % of trips), these participants were not categorized into a specific mode of commuting, and therefore classified as “not categorized” and used as a reference population in statistical analyses [45].

### Socio-demographic characteristic

The socio-demographic variables assessed included gender, educational level and ethnicity. Socioeconomic status (SES) was measure using one item: “How many years of education have you completed?” (low: no college or university education/high: having attended college or university). Ethnicity was obtained from the children’s questionnaire, determined by two questions regarding the parents’ native country: “What is your mother native country?” and “What is your father native country?” The parents who respond to the questionnaire (mother or father) were categorized into two groups: native Norwegians (born in Norway) and not native Norwegians (not born in Norway).

### Personal and environmental characteristics

Access to car, bicycle, car parking at work was assessed using three items; “Do you have a car for personal use?”; Do you have a bike for personal use?”; “Do you have access to car parking at work?”. Items were rated yes/no. Moreover, the responders reported number of cars for personal use. Items were rated no car (0), one car (1) and more than one car (2). Regarding perceptions about traffic safety, parents were asked to “Rank the level of road safety on your way to your workplace from 1 (very dangerous) to 5 (completely safe)”. Personal attitudes regarding active transport and car use to work was accessed by the following statements; “I like to walk or cycle to work”; “I use the way to work as exercise to keep myself in good physical shape”; “I rarely walk or cycle to and from work if the weather is bad”; “In terms of travel choice I always choose the most environmentally friendly ways of traveling”; “I limit my car use to reduce CO2 emissions” and “I always use the car when grocery shopping”. The answers response was collapsed into two categories into a median cut of in order to reduce the number of single variables.

### Behavioral characteristics

Leisure time physical activity was asses using two items; “Do you exercise regularly?” (response option was yes/no) and “How many times a week do you exercise to the extent that you experience shortness of breath and/or sweating?” A number of six response alternatives was rated from “every day” = 1 to “never” = 6. Based on the answers, leisure time physical activity was subsequently categorized into “low” (once a week or less), and “high” (2–3 times a week or more) based on a median cut. Regarding sedentary behavior, parents were asked “How many hours per day during leisure time do you usually watch TV and/or sit in front of your computer?” Items was rated from “never” = 1 to “more than 4 h” = 6. Parents who reported ½–1 h or less were categorized into “low” and those reported 2–3 h or more were categorized into “high” degree of sedentary behavior. Sleeping hours was reported using one item; “How many hours of sleep do you usually get at night?” Item was dichotomized into “less than 7 h” and “7 h or more”.

### Distance to work, weight status, age, and gender

Perceived distance in kilometers between home and workplace was provided by the questionnaire. Two dichotomous variables were created: living less or more than 3 km from work, or living less or more than 5 km from work. The relationship between commuting distance and choice of mode is unclear [13, 19], so we choose to conduct distance cut-offs based on subjective values from different experiences. The cut offs were used in the statistical analysis for walking (3 km) and cycling (5 km) and driving (5 km), respectively. Age was calculated based on date of birth. Body Mass Index (BMI, kg/m^2^) was calculated from self-reported values of height and weight and overweight defined as a BMI above 25.

### Statistical analysis

In descriptive analyses, the responders were grouped in to their respective modes of commuting and the unadjusted relationships with potential correlates were assessed with one-way analysis of variance (ANOVA) and chi-squared test. In adjusted analyses, we used multivariate logistic regression to identify potential correlates associated with the probability of being either walker (vs. non-walkers), cyclist (vs. non cyclists) or car commuter (vs. non-car commuters). We did not assess the correlates of public transportation due to few participants categorized into this mode of commuting (*n* = 17). Walking, cycling and non-active commuters were first compared to the rest of the sample (eg walkers, were compare to non-walkers (ie cyclists, non-active commuters and parents not categorized into mode of commuting) and then, walkers and non-active commuters were compared to cyclist).

Independent variables were included in the final multivariate models if they were statistically significant at (*p* < 0.05) in the univariate analysis. In model 1, the socio-demographic variables were included. Model 2 further included variables related to personal attitudes and environmental factors. Model 3 further included variables related to the selected health-related behaviors. The results of the logistic regression are given as odds ratios (OR) with 95 % confidence intervals (95 % CI). The level of statistical significance was *p* < 0.05. All the statistical analyses were performed using SPSS 20.0 for windows (SPSS Inc., Chicago, IL).

## Results

Descriptive data are presented in Table 1 and shows that the proportions of the participants categorized as walkers, cyclist, car and public transport commuters were 7.3, 12.3, 70.4 and 2.4 %, respectively. A total of 7.6 % did not meet the criteria to be categorized into any modes of transport. Gender was reports as male or female, and we did not find any significant associations among gender differences and commuting mode (Table 1). Characteristics between individuals with complete data (*n* = 709) and those who were excluded (*n* = 303) with respect gender, education, regularly exercise, distance to work and mode of commuting to work were tested by oneway Anova.

**Table 1:** Description of mode of commuting and the unadjusted association between mode of commuting and socio demographics collected among Norwegian parents

			Walkers		Cyclist		Car commuters		Public transporters	
	*n*	%	%	(95 % CI)	%	(95 % CI)	%	(95 % CI)	%	(95 % CI)
Total sample	709	100.0	7.3	−	12.3	−	70.4	−	2.4	−
Male	157	22.8	6.3	(2.5–10.2)	12.1	(6.9–17.2)	7.3	(6.5–8.0)	5.1	(1.6–8.6)
Female	533	77.7	7.7	(5.4–10.0)	12.2	(9.1–14.9)	6.9	(6.5–7.3)	1.7	(0.5–2.8)
Low education	291	41.2	5.2	(2.6–7.7)	13.1	(9.2–17.0)	7.0	(6.5–7.6)	3.1	(1.1–5.1)
High education	416	58.8	8.9	(6.2–11.6)	11.5	(8.5–14.7)	7.1	(6.6–7.5)	1.9	(0.1–3.3)
Regular exercise	445	63.6	6.7	(4.4–9.1)	15.3^a^	(11.9–18.7)	6.7	(6.2–7.1)	2.0	(0.7–3.3)
Not regular exercise	255	36.4	8.6	(5.2–12.1)	7.1	(3.9–10.2)	7.7^a^	(7.2–8.2)	3.1	(0.9–5.2)
Distance less than 3 km	202	29.1	19.3	(13.8–24.8)	27.2^a^	(21.0–33.4)	37.1	(30.4-43.9)	0.9	(−0.4–2.4)
Distance more than 3 km	492	70.9	2.4	(1.1–3.8)	6.1	(4.0–8.2)	83.9^a^	(80.7–87.2)	3.1	(1.5–4.6)

^a^Significant difference between groups (chi-square statistics, *P* > 0.05)

Overall, the study population was mostly ethnic Norwegians (93.9 %), females (77.2 %), and had a high educational level (58.8 %). The mean commuting distance to the workplace was 3.2 km ±22.5 and mean age of respondents was 41.7 ± 5.3 years. A total of 63 % of the study population reported to exercise regularly. Distance from home to work was strongly associated with mode of commuting. Those living less than 3 km from work were more likely to be categorized as a cyclist, whereas those living more than 3 km from work were more likely to be categorized as car commuters (Table 1). Table 2 shows that parents were more likely to be a walker if the distance to work was less than 3 km (19.3 vs. 2.4 %, *OR* = 4.6, 95 % *CI* = 2.0-10.7), if the traffic was considered to be safe (*OR* = 1.2 for each incremental increase in perceived traffic safety, 95 % *CI* = 1.0-1.5), and if they had a positive attitude towards reducing car CO_2_ emissions (12.6 vs. 5.7 %, *OR* = 2.1, 95 % *CI* = 1.0 – 4.7). Parents were less likely to be a walker if they had access to more than one car (3.9 vs. 49.2 %, *OR* =0.2, 95 % *CI* =0.0, 0.8) or if they considered weather as an obstacles for active commuting (19.9 vs. 2.9 %, *OR* = 0.3, 95 % *CI* = 0.1–0.6).

**Table 2:** Correlates of walking to and from work (*n* = 52) in comparison to not walking (*n* = 657)

Variable	Model 1^a^		Model 2^b^	
	OR	95 % CI	OR	95 % CI
Distance (less than 3 km)	9.6	(4.9, 19)	4.6	(2.0, 11)
Access to car				
One car			0.3	(0.1,1.2)
More than one car			0.2	(0.0,0.8)
Access to car park			0.5	(0.2, 1.2)
Traffic safety (scale 1–10)			1.2	(1.0, 1.5)
Positive attitude towards walking or cycling to work			4.5	(0.9, 22)
Regards commuting as exercise			1.1	(0.5, 2.5)
Regards weather as obstacle to active commuting			0.3	(0.1, 0.6)
Limits car use to reduce C02 emissions			2.1	(1.0, 4.7)
Positive attitude towards environmentally ways of traveling			0.8	(0.4, 1.7)
Use car for grocery shopping			1.8	(0.8, 4.1)

^a^Based on multivariate logistic regression analysis

^b^In model 2, there were 554 participants due to missing information on covariates (*n* = 155)

Table 3 shows the results of the multivariate logistic regression assessing the probability of being a cyclist. Similar to walkers, the parents were more likely to be a cyclist if the distance to work was less than 5 km (22.1 vs. 3.3 %, *OR* = 3.0 = 95 % *CI* = 1.4–6.3) and if the surrounding traffic was perceived as safe (*OR* = 1.1, 95 % *CI* = 1.0–1.3) and less likely if the weather was considered to be an obstacle (2.9 vs. 19.9 %, *OR* = 0.2 = 95 % *C1* = 0.1–0.4). Additionally, parents who considered cycling to work as exercise to maintain physical shape was more likely to be cyclists (15.3 vs. 7.1 %, *OR* = 2.7, 95 % *CI* = 1.4–5.4) while those using the car for grocery shopping was less likely to be cyclist (5.9 vs. 11.7 %, *OR* = 0.4, 95 % *CI* = 0.2–0.8).

**Table 3:** Correlates of cycling to and from work (*n* = 87) in comparison to not walking (*n* = 622)

Variable	Model 1^a^		Model 2		Model 3^b^	
	OR	95 % CI	OR	95 % CI	OR	95 % CI
Distance (less than 5 km)	8.3	(4.4, 15.7)	2.9	(1.4, 6.0)	3.0	(1.4, 6.3)
Own a bike			3.4	(0.7,16.8)	3.0	(0.6, 15.3)
Access to car						
One car			1.9	(0.5, 7.0)	1.8	(0.5, 7.0)
More than one car			1.4	(0.3, 5.7)	1.4	(0.3, 5.6)
Traffic safety (scale 1–10)			1.1	(1.0, 1.3)	1.1	(1.0, 1.3)
Positive attitude towards walking or cycling to work			1.7	(0.6, 4.7)	1.6	(0.6, 4.6)
Regards commuting as exercise			2.8	(1.4, 5.6)	2.7	(1.4, 5.4)
Regards weather as obstacle to active commuting			0.2	(0.1, 0.4)	0.2	(0.1, 0.4)
Limits car use to reduce C02 emissions			1.5	(0.8, 2.8)	1.5	(0.8, 2.8)
Positive attitude towards environmentally ways of traveling			1.2	(0.7, 2.2)	1.2	(0.6, 2.2)
Use car for grocery shopping			0.4	(0.2, 0.8)	0.4	(0.2, 0.8)
Performs physical training regularly					1.4	(0.7, 2.8)

^a^Based on multivariate logistic regression analysis. ^b^In model 3, there were 543 participants due to missing information on covariates (*n* = 166)

Table 4 shows the results of the multivariate logistic regression assessing the probability of being a car commuter. Parents were more likely to be a car commuter if they considered weather as an obstacle (82.5 vs. 29.6 %, *OR* = 8.7, 95 % *CI* = 4.6–16.4) and if they used car for grocery shopping (78.8 vs. 45.0 %, *OR* = 2.2, 95 % *CI* = 1.2–4.1). Parents were less likely commute by car if the distance to work was below 5 km (48.2 vs. 90.4 %, *OR* = 0.1, 95 % *CI* = 0.1–0.3), if they perceived the traffic to be safe (*OR* = 0.9 for each incremental decrease in perceived traffic safety, 95 % *CI* = 0.8–1.0), if they considered commuting as an opportunity to exercise (39.7 vs. 83.7 %, *OR* = 0.3, 95 % *CI* = 0.1–0.5) and if they tried to limit car use in order to reduce CO2 emissions (53.7 vs. 76.0 %, *OR* = 0.5, 95 % *CI* = 0.3–1.0). Additionally, parents were less likely to be car commuters if they only had access to one car instead of more than one (55.9 vs. 83.1 %, *OR* = 0.4, 95 % *CI* = 0.2–08) and if they were of non-native Norwegian ethnicity (50.0 vs. 71.9 %, *OR* = 0.1, 95 % *CI* = 0.0–0.5).

**Table 4:** Correlates of driving to and from work (*n* = 499) in comparison to not walking (*n* = 210)

Variable	Model 1^a^		Model 2		Model 3^b^	
	OR	95 % CI	OR	95 % CI	OR	95 % CI
Native Norwegian	0.3	(0.1, 0.5)	0.1	(0.0, 0.5)	0.1	(0.0, 0.5)
Distance (less than 5 km)	0.1	(0.1, 0.1)	0.1	(0.1, 0.3)	0.1	(0.1, 0.3)
Own a bike			0.4	(0.1, 1.3)	0.4	(0.1, 1.3)
Access to car (reference = one car)						
More than one			0.4	(0.2, 0.7)	0.4	(0.2, 0.8)
Access to car park			3.3	(1.0, 10.5)	3.1	(1.0, 10)
Traffic safety (scale 1–10)			0.9	(0.8, 1.0)	0.9	(0.8, 1.0)
Positive attitude towards walking or cycling to work			0.5	(0.2, 1.0)	0.5	(0.2, 1.0)
Regards commuting as exercise			0.2	(0.1, 0.5)	0.3	(0.1, 0.5)
Regards weather as obstacle to active commuting			8.4	(4.5, 16)	8.7	(4.6, 16)
Limits car use to reduce C02 emissions			0.5	(0.3, 1.0)	0.5	(0.3, 1.0)
Positive attitude towards environmentally ways of traveling			0.5	(0.3, 1.0)	0.6	(0.3, 1.0)
Use car for grocery shopping			2.2	(1.2, 4.1)	2.2	(1.2, 4.1)
Performs physical training regularly					0.8	(0.3, 1.7)
Low levels of leisure time physical activity					0.8	(0.5, 1.7)

^a^Based on multivariate logistic regression analysis

^b^In model 3, there were 524 participants due to missing information on covariates (*n* = 185)

## Discussion

We have described the associations between modes of commuting to the workplace among parental adults by taking socio-demographic, personal, environmental and behavioral factors into account. We found several correlates associated with being either a walker, cyclist or car commuter. Consistent with others studies [20, 22, 36, 47, 48], we found that commuters with shorter distance between home and workplace were more likely to be walkers and cyclists, and we also found them to be less likely to commute by car. Higher levels of perceived traffic safety were associated with increased probability of walking and cycling, and slightly decreased probability of commuting by car. This is in line with findings from other studies; which have reported about a positive association between perceived traffic safety and the probability of walking or cycling to work [31, 49, 50]. Moreover, infrastructural initiatives through urban design of land use and planning at community, street scales and active transport policy have been found as effective practices to increase active commuting [31, 36, 47].

In our study, data showed that those who commuting by car reported slightly lower traffic safety on the way to work compared to walkers and cyclists. Drivers may have to deal with stressful situations due to high traffic stream and vehicular queuing, which might lead to them feeling less safe in traffic and hence explain the association between driving a car and feeling less safe in traffic. Research study traffic safety has found that lack of control in traffic situations can promote stress among drivers [30]. On the other hand, parents who experience car commuting as less safe may be more inclined to change behavior and be a potential target for campaigns promoting active commuting. We also found that parents were more likely to be car commuters if they had access to more than one car. The reason for this could be that parents might find it more convenient to use the car when it is readily available. It is no doubt that there is a global need to reduce climate gas emissions and motorization, which demands initiative and raising obviously important questions for the future well-being around the world [51–53]. There are several practicalities in everyday life that may influence modes of transport. Norway is a country with cold climate and shifting weather that may discourage people from doing outdoors activities. We found that attitudes towards active commuting in bad weather were associated with reduced probability of walking and cycling and increased probability of car commuting. This association has also been reported in populations from US and Austria [49, 54, 55]. Weather is an obstacle that cannot be removed, but the impact may be reduced if bike paths and sidewalks are kept free from snow and ice during wintertime and by providing people access to adequate facilities at work, such as wardrobes with showers and lockers. Moreover, grocery shopping may also be more convenient with a car due to the difficulties of carrying heavy loads; hence, we found that those who reported using the car for grocery shopping, were more likely to be car commuters and less likely cycle. Some research has suggested that environmental concern and health benefits are associated with active commuting [54–56]. We found that parents with a positive attitude towards reducing CO_2_ emissions were more likely to be walkers, but less likely to be car commuters. Further, we found those who considered travelling to work as an opportunity to maintain physical health, were more likely to be a cyclist and less likely to be a car commuter. Increased awareness and knowledge regarding the environmental and health benefits of active transportation may be an important strategy in the promotion of active transportation. The results of this study showed that the prevalence of walking (7.3 %) was somewhat lower compared to estimates based on the total Norwegian population (11 %) [41]. Since data was collected from two counties in Norway, not surrounded by any large cities, the difference may therefore be due to longer distances between home and work compared to what might be the case in larger cities. In contrast, the proportion that cycled to work was higher (12.3 % compared to 6 %), suggesting that cycling may be both feasible and preferable to walking when actively commuting in this geographical region. As expected, fewer people used public transportation to work in our study than what have been observed in the total Norwegian population (2.4 and 15 %, respectively). On the other hand, more people drove car (70 % compared to 61 %, respectively). There is limited availability of public transportation in our study area compared to large cities and this might lead to an increase in the need to drive cars. In light of these findings, cycling to work seems to be the most appropriate target for interventions and public health campaigns within this population. Henceforth, this study is a contribution to the research field in order to facilitate the social and environmental condition to active commuting so walking and cycling substitute car trips as the default choice in order to improve public health in all segments of the population.

### The strengths and limitations

The main strength of this study is the large sample and the precision of the measurement of active commuting as the main exposure and multiple measures of outcome variables. We used a reliable comprehensive self-reported design of the measure on commuting to work, making it possible to assess the frequency of the different modes of active commuting to and from the workplace [26, 45]. Since there has been little agreement on how commuting should be measured, inconsistent measures of travel habits have been reported in previous studies with little or no information on their validity and reliability.

However, a key limitation of data collected by self-reporting questionnaire that could have affected the results is that participants may answer differently about the frequency of active commuting in order to adhere to social norms regarding physical activity and health lifestyle. In addition, the cross-sectional design of this study makes it impossible to draw conclusions regarding specific causal relationship between active commuting, determinants and personal barriers. A total of 1 912 parents were eligible invited to take part in the study, were only 709 were considered in the analysis. This may probably have resulted in a significant bias in results. Furthermore, more mothers than fathers respond to the questionnaire, and this raising question about the generalizability. Ideally, gender responders should have been evenly distributed in the study. We also found a small numbers of participants walking and cycling and this is clearly a limitation when analyzing the associations’ factors.

On the contrary, we used perceived distance between home and workplace and this may be different from objective measured distance. In addition, some of the observed relationships between individual modes of transport and correlates may also not necessarily be generalizable to other populations, such as those from more urban areas. However, the association between active transportation and perceived health benefits and environmental concern should be valid for many commuters.

## Conclusion

In this cross-sectional study of parents living in sub-urban Norway, we found that active commuting to and from the workplace were associated with a shorter distance to work, traffic safety, environmental concern, health benefits and weather condition.

The authors recommend further research studies to examine the effect of social interaction between parents and children in addition to school and community involvements, and addressing the complexity of multiple factors influencing active commuting. Parents may have unique challenges to face as a role model of social and spousal support. For public and environmental health, more knowledge about commuting habits is important and necessary to identify effective models for using evidence in the policy making process. Public health strategies should encourage a high level of active commuting and provide a bike and walking-friendly environment that supports active commuting, in order to tackle triple challenges of health issues in the future.

## References

[1] Heath GW, Parra DC, Sarmiento OL, Andersen LB, Owen N, Goenka S, Montes F, Brownson RC (2012). Evidence-based intervention in physical activity: lessons from around the world. Lancet.

[2] Lee I, Shiroma EJ, Lobelo F, Puska P, Blair SN, Katzmarzyk PT (2012). Effect of physical inactivity on major non-communicable diseases worldwide: an analysis of burden of disease and life expectancy. Lancet.

[3] Pratt M, Norris J, Lobelo F, Roux L, Wang G (2014). The cost of physical inactivity: moving into the 21st century. Br J Sports Med.

[4] Andersen LB, Schnohr P, Schroll M, Hein HO (2000). All-cause mortality associated with physical activity during leisure time, work, sports, and cycling to work. Arch Intern Med.

[5] Anokye NK, Trueman P, Green C, Pavey TG, Taylor RS (2012). Physical activity and health related quality of life. BMC Public Health.

[6] Brown DR, Carroll DD, Workman LM, Carlson SA, Brown DW (2014). Physical activity and health-related quality of life: US adults with and without limitations. Qual Life Res.

[7] Penedo FJ, Dahn JR (2005). Exercise and well-being: a review of mental and physical health benefits associated with physical activity. Curr Opin Psychiatry.

[8] Bauman AE, Reis RS, Sallis JF, Wells JC, Loos RJ, Martin BW (2012). Correlates of physical activity: why are some people physically active and others not?. Lancet.

[9] Aldred R (2014). Promoting walking and cycling: New perspectives on sustainable travel. Transp Rev.

[10] Oja P, Vuori I, Paronen O (1998). Daily walking and cycling to work: their utility as health-enhancing physical activity. Patient Educ Couns.

[11] Hamer M, Chida Y (2008). Active commuting and cardiovascular risk: a meta-analytic review. Prev Med.

[12] Pucher J, Buehler R, Bassett DR, Dannenberg AL (2010). Walking and cycling to health: a comparative analysis of city, state, and international data. Am J Public Health.

[13] Shephard R (2008). Is active commuting the answer to population health?. Sports Med.

[14] Bassett D, Pucher J, Buehler R, Thompson D, Crouter S (2011). Active transportation and obesity in Europe, north America, and Australia. ITE J.

[15] Mytton OT, Panter J, Ogilvie D (2016). Longitudinal associations of active commuting with body mass index. Prev Med.

[16] Cooper AR, Wedderkopp N, Jago R, Kristensen PL, Moller NC, Froberg K, Page AS, Andersen LB (2008). Longitudinal associations of cycling to school with adolescent fitness. Prev Med.

[17] Andersen LB, Froberg K (2015). Advancing the understanding of physical activity and cardiovascular risk factors in children: the European Youth Heart Study (EYHS). Br J Sports Med.

[18] Børrestad LAB, Østergaard L, Andersen LB, Bere E (2012). Experiences from a randomised, controlled trial on cycling to school: Does cycling increase cardiorespiratory fitness?. Scand J Public Health.

[19] Hallal PC, Andersen LB, Bull FC, Guthold R, Haskell W, Ekelund U (2012). Global physical activity levels: surveillance progress, pitfalls, and prospects. Lancet.

[20] Cohen A, Ross Anderson H, Ostro B, Pandey K, Krzyzanowski M, Künzli N, Gutschmidt K, Pope A, Romieu I, Samet J, Smith K (2005). The global burden of disease due to outdoor air pollution. J Toxicol Environ Health A.

[21] Gatersleben B, Uzzell D (2007). Affective appraisals of the daily commute: comparing perceptions of drivers, cyclists, walkers, and users of public transport. Environ Behav.

[22] Willis DP, Manaugh K, El-Geneidy A (2015). Cycling under influence: summarizing the influence of perceptions, attitudes, habits, and social environments on cycling for transportation. Int J Sustain Transp.

[23] Heinen E, Van Wee B, Maat K (2010). Commuting by bicycle: an overview of the literature. Transp Rev.

[24] Bassett DR, Pucher J, Buehler R, Thompson DL, Crouter SE (2008). Walking, cycling, and obesity rates in Europe, North America and Australia. J Phys Act Health.

[25] Motte B, Aguilera A, Bonin O, Nassi CD (2016). Commuting patterns in the metropolitan region of Rio de Janeiro. What differences between formal and informal jobs?. J Transp Geogr.

[26] Cole-Hunter T, Donaire-Gonzalez D, Curto A, Ambros A, Valentin A, Garcia-Aymerich J, Martínez D, Braun LM, Mendez M, Jerrett M, Rodriguez D, de Nazelle A, Nieuwenhuijsen M (2015). Objective correlates and determinants of bicycle commuting propensity in an urban environment. Transp Res Part D: Transp Environ.

[27] Pucher J, Dijkstra L (2003). Promoting safe walking and cycling to improve public health: lessons from The Netherlands and Germany. Am J Public Health.

[28] Vandenbulcke G, Dujardin C, Thomas I, Geus BD, Degraeuwe B, Meeusen R, Panis LI (2011). Cycle commuting in Belgium: Spatial determinants and ‘re-cycling’ strategies. Transp Res A Policy Pract.

[29] Christiansen LB, Christiansen LB, Madsen T, Schipperijn J, Ersbøll AK, Troelsen J (2014). Variations in active transport behavior among different neighborhoods and across adult life stages. J Trans Health.

[30] Vågane L, Brechan I, Hjorthol R. Transport volumes in Norway 1946–2012. Transport economic institute, Report: 1277/2013. Oslo; 2013. p.42.

[31] Pucher J, Buehler R (2008). Making cycling irresistible: lessons from the Netherlands, Denmark and Germany. Transp Rev.

[32] Buehler R (2012). Determinants of bicycle commuting in the Washington, DC region: The role of bicycle parking, cyclist showers, and free car parking at work. Transp Res Part D: Transp Environ.

[33] de Geus B, De Bourdeaudhuij I, Jannes C, Meeusen R (2007). Psychosocial and environmental factors associated with cycling for transport among a working population. Health Educ Res.

[34] Børrestad LAB, Andersen LB, Bere E (2011). Seasonal and socio- demographic determinants of school commuting. Prev Med.

[35] de Bruijn G, Kremers SPJ, Singh A, van den Putte B, van Mechelen W (2009). Adult active transportation: adding habit strength to the theory of planned behavior. Am J Prev Med.

[36] Panter JR, Jones AP, van Sluijs EMF, Griffin SJ, Wareham NJ (2011). Environmental and psychological correlates of older adult's active commuting. Med Sci Sports Exerc.

[37] Lemieux M, Godin G (2009). How well do cognitive and environmental variables predict active commuting?. Int J Behav Nutr Phys Act.

[38] Craig CL, Brownson RC, Cragg SE, Dunn AL (2002). Exploring the effect of the environment on physical activity: A study examining walking to work. Am J Prev Med.

[39] Bopp M, Behrens TK, Velecina R (2014). Associations of weight status, social factors, and active travel among college students. Am J Health Educ.

[40] Campbell MEBM (2013). An examination of the relationship of interpersonal influences with walking and biking to work. J Public Health Manag Pract.

[41] Ulf E, Daniel A, Klaus G, Henrik O, Kristina S (2012). Walkability parameters, active transportation and objective physical activity: moderating and mediating effects of motor vehicle ownership in a cross- sectional study. Int J Behav Nutr Phys Act.

[42] Yang L, Panter J, Griffin SJ, Ogilvie D (2012). Associations between active commuting and physical activity in working adults: Cross-sectional results from the Commuting and Health in Cambridge study. Prev Med.

[43] Badland HM, Schofield GM, Garrett N (2008). Travel behavior and objectively measured urban design variables: Associations for adults traveling to work. Health Place.

[44] Bere E, Hilsen M, Klepp K (2010). Effect of the nationwide free school fruit scheme in Norway. Br J Nutr.

[45] Bere E, Bjørkelund LA (2009). Test-retest reliability of a new self reported comprehensive questionnaire measuring frequencies of different modes of adolescents commuting to school and their parents commuting to work - The ATN questionnaire. Int J Behav Nutr Phys Act.

[46] Borrestad L, Ostergaard L, Andersen L, Bere E (2013). Associations between active commuting to school and objectively measured physical activity. J Phys Act Health.

[47] Titze S, Stronegger W, Janschitz S, Oja P (2008). Association of built-environment, social-environment and personal factors with bicycling as a mode of transportation among Austrian city dwellers. Prev Med.

[48] Engbers LH, Hendriksen IJM (2010). Characteristics of a population of commuter cyclists in the Netherlands: perceived barriers and facilitators in the personal, social and physical environment. Int J Behav Nutr Phys Act.

[49] Bopp M, Child S, Campbell M (2014). Factors associated with active commuting to work among women. Women Health.

[50] Guell C, Panter J, Ogilvie D (2013). Walking and cycling to work despite reporting an unsupportive environment: insights from a mixed-method exploration of counterintuitive findings. BMC Public Health.

[51] Pucher J, Dill J, Handy S (2010). Infrastructure, programs, and policies to increase bicycling: An international review. Prev Med.

[52] Ogilvie D (2004). Promoting walking and cycling as an alternative to using cars: Author's reply. BMJ.

[53] Pucher J, Peng Z, Mittal N, Zhu Y, Korattyswaroopam N (2007). Urban transport trends and policies in china and India: impacts of rapid economic growth. Transp Rev.

[54] Kaczynski A, Bopp M, Wittman P (2012). To drive or not to drive: factors differentiating active versus non-active commuters. J Health Behav Publ Health.

[55] Zhang H (2013). Neighbourhood, route and workplace- related environmental characteristics predict Adults’ mode of travel to work. PLoS One.

[56] Pratt M, Sarmiento OL, Montes F, Ogilvie D, Marcus BH, Perez LG, Brownson RC (2012). The implications of megatrends in information and communication technology and transportation for changes in global physical activity. Lancet.

